# Supplementary material for: Extended-spectrum β-lactamase-producing Escherichia coli isolated from captive primates: characteristics and horizontal gene transfer ability analysis
Source: PLoS One. 2025 Apr 11;20(4):e0321514. doi: 10.1371/journal.pone.0321514 (PMC11990791; doi:10.1371/journal.pone.0321514)
Supplement: Attachment — Submitted filename: Response to Reviewers.docx [file pone.0321514.s005.docx]

Dear Editor:

Thank you very much for your letter and the reviewers’ comments regarding our manuscript submitted to “PLOS ONE” (Manuscript ID: PONE-D-24-55572). We have checked the article and revised it according to the comments, and carefully proof-read the manuscript. The language presentation was improved with assistance from English speakers with appropriate research background. All revisions were marked in revised manuscript with track changes.

We hope, with these modifications and improvements based on your suggestion and the reviewer’s comments, the quality of our manuscript would meet the publication standard of “PLOS ONE”. Once again, we acknowledge your comments and constructive suggestions very much, which are valuable in improving the quality of our manuscript.

We submit here the revised manuscript with track changes as well as a list of changes. Thanks again for your reconsideration of our manuscript for publication in your journal. If you have any question about this paper, please don’t hesitate to let me know.

Best wishes for you!

Sincerely yours, Zhijun Zhong

College of Veterinary Medicine

Key Laboratory of Animal Disease and Human Health of Sichuan Province

Sichuan Agricultural University

Chengdu 611130, P. R. China

E-mail: zhongzhijun488@126.com

**Response to Reviewer 1 Comments**

**Response to Reviewer:**

Thank you for reading the manuscript carefully and providing many thoughtful comments, which will significantly improve the quality of the manuscript. We have carefully revised the manuscript in accordance with your comments (Tracked Version). Detailed responses are as follows:

**Reviewer #1**: This manuscript describes the characteristics of Escherichia coli isolated from captive primates. The objective is clear, and the methods used are appropriate. While the results offer some value to readers in this specific field, they do not necessarily present novel findings. I provide the following editorial comments and questions to help improve the readability of the manuscript. Additionally, I strongly recommend that the authors have their revised manuscript undergo thorough English editing before resubmission

Thank you for your suggestions about the language of the manuscript, we carefully checked the English expression and made English revisions under the scrutiny of native English speakers with appropriate research background.

**Comment 1:** Line 29: remove E. coli

**Response 1:** Thanks for your comments, we have removed *E. coli* in our revised manuscript (*Escherichia coli* is a resident microorganism in the gut of humans and…) (Tracked Version Line 40)*.*

**Comment 2:** Line 30: Replace all instances of Escherichia coli with E. coli as an abbreviation throughout the entire manuscript.

**Response 2:** Thanks for your comments, we have checked the abbreviation error and corrected Escherichia coli to *E. coli* in our revised manuscript.

**Comment 3:** Line 33: see comment in line 30.

**Response 3:** Thanks for your comments, we have corrected the error in our revised manuscript (The ESBL-producing *E. coli* (ESBL-EC) is one of the most important pathogens at the One Health interface…) (Tracked Version Line 45).

**Comment 4:** Line 46: Remove one occurrence of 'captive' as it is repeated.

**Response 4:** Thanks for your comments, we have removed the repeated 'captive' in our revised manuscript (In 2024, Bazalar-Gonzales et al [20] isolated seven ESBL-EC strains from captive and semi-captive primates, three *bla*_CTM-X_ variants...) (Tracked Version Line 62).

**Comment 5:** Line 76: E. coli should be italicized. This is an incomplete sentence.

**Response 5:** Thanks for your comments, we have corrected the unitalicized error, checked the grammar and revised the sentence to make the sentence structure complete as follow: ‘Four hundred and forty-four MDR *E. coli* strains from captive primates, which were isolated from 13 zoos and had previously been stored in our laboratory, were used in this study’ (Tracked Version Line 95-97).

**Comment 6:** Line 93: please check irp2 or Irp2 here.

**Response 6:** Thanks for your comments. After checking, we confirmed that it was *Irp2* and corrected the error in our revised manuscript.

**Comment 7:** Line 104: used as the recipient strain

**Response 7:** Thanks for your comments, we have corrected the error in our revised manuscript (In this study, the sodium azide-resistant *E. coli* J53 was used as the recipient strain to evaluate the conjugation and transfer ability of…) (Tracked Version Line 129).

**Comment 8:** Line 105: Those colonies capable….

**Response 8:** Thanks for your comments, we have added the word to make the sentence correct (Those colonies capable of growing on Luria-Bertani (LB) agar supplemented with CTX (4 mg/mL) and sodium aside (100 mg/mL) were…) (Tracked Version Line 130).

**Comment 9:** Line 121: This is an incomplete sentence.

**Response 9:** Thanks for your comments, we carefully checked the sentences for grammatical errors and corrected them as follows: ‘Phylogenetic analysis (A, B1, B2, D) was conducted by detecting *yjaA*, *chuA*, and *TSPE4.C2*, according to Clermont et al’ (Tracked Version Line 150-151).

**Comment 10:** Line 131: Replace the comma (,) with a semicolon (;).

**Response 10:** Thank you for pointing out the error, we have corrected it in our revised manuscript (…VAGs were performed using R-software (version 4.2.2) [40]; *P-value...*) (Tracked Version Line 163).

**Comment 11:** Line 144-148: You might consider rephrasing it as: Among the five zoos, the most prevalent gene combination pattern in Guiyang Zoo was blaCTX-M-15 (32.00%, 8/25); in Hangzhou Zoo, it was blaCTX-M-55 & blaTEM-198 (34.78%, 8/23); in Chongqing Zoo, it was blaCTX-M-15 (25.00%, 3/12); in Dalian Zoo, it was blaCTX-M-15 (37.50%, 3/8); and in Changsha Zoo, it was blaCTX-M-55 (100%, 1/1).

**Response 11:** Thank you for the detailed suggestions for revision. We have revised the sentence accordingly in our revised manuscript (Among the five Zoos, the most prevalent gene combination pattern in Guiyang Zoo was *bla*_CTX-M-15_ (32.00%, 8/25), in Hangzhou Zoo, it was *bla*_CTX-M-55_ & *bla*_TEM-198_ (34.78%, 8/23), in Chongqing Zoo, it was *bla*_CTX-M-15_ (25.00%, 3/12), in Dalian Zoo, it was *bla*_CTX-M-15_ (37.50%, 3/8), and in Changsha Zoo, it was *bla*_CTX-M-55_ (100.00%, 1/1).) (Tracked Version Line 183-186).

**Comment12:** Line 157: replace greatest with largest

**Response 12:** Thanks for your comments. We have revised this sentence in our revised manuscript.

**Comment 13:** Line 205-206: Please explain how ESBLs produced by Enterobacteriaceae are ineffective against β-lactam drugs? Do you mean “β-lactam drugs are ineffective against ESBLs produced by Enterobacteriaceae”?

**Response 13:** Thank you for your comment, ESBLs are produced by some bacteria such as *Enterobacteriaceae* and are able to hydrolyze β-lactam antibiotics, thus leading to bacterial resistance to these antibiotics. Sorry for the wrong expression here, we have corrected the sentence as follows: "β-lactams are the most prescribed antibiotics worldwide. However, β-lactams antibiotics are ineffective against ESBL-producing *Enterobacteriaceae.*" (Tracked Version Line 253)

**Comment 14:** Line 211: Only two categories of ESBLs-genes…

**Response 14:** Thank you for your comment, we have corrected it in our revised manuscript (Only two categories of ESBL genes (*bla*_CTX-M_ and *bla*_TEM_) were…) (Tracked Version Line 259).

**Comment 15:** Line 216: in a variety of animals

**Response 15:** Thank you for your comment, we have corrected it in our revised manuscript (*bla*_CTX-M-55_ is being increasingly detected in a variety of animals, including waterfowl…) (Tracked Version Line 269).

**Comment 16:** Line 246-248: You might consider rephrasing it as: The combination of ESBL production and the carriage of virulence-associated genes (VAGs) in E. coli will undoubtedly limit therapeutic options, increase treatment complexity, and enhance pathogenicity.

**Response 16:** Thank you for your comments. Your opinions make the expression more scientific and rigorous. We have revised it according to your suggestions in our revised manuscript (Tracked Version Line 305-307).

**Comment17:** Line 248-250: You might consider rephrasing it as: Additionally, strains that carry various VAGs may not be harmful to animals, but there is a potential for them to become pathogenic if host immunity weakens or in response to other adverse environmental factors.

**Response 17:** Thank you for your comments. We have revised the sentence in our revised manuscript (Tracked Version Line 310-312).

**Comment 18:** Line 262: The Trbc protein

**Response 18:** Thanks for pointing out the error, we have corrected it in our revised manuscript (The TrbC protein plays an important role in conjugative transfer…) (Tracked Version Line 330).

**Comment 19:** Line 267-269: You might consider rephrasing it as: The plasmid incompatibility groups detected in our study included IncFIB, IncFrepB, IncI1, IncFIA, IncHI1, and IncP.

**Response 19:** Thank you for your comments, we have revised the sentence in our revised manuscript (Tracked Version Line 337).

**Comment 20:** Line 283-285: You might consider rephrasing it as: Moreover, plasmids, particularly IncF plasmids, have been reported as the most common vehicles for the transmission of VAGs. Our study demonstrated that 9 VAGs can be transferred into the recipient strain J53 through conjugation with plasmids.

**Response 20:** Thank you for your comments. We have improved the sentence according to the suggestions you provided in our revised manuscript (Tracked Version Line 355-358).

**Comment 21:** Line 296-300: The ST detected in this study requires a more detailed discussion.

**Response 21:** Thank you for your comments and we have discussed the detected STs in more detail in our revised manuscript as follow: ‘Moreover, ST533 was previously detected in canine ESBL-EC isolates (harboring the *bla*_CTX-M-15_ gene) [73], ST4358 was identified in *E. coli* isolated from goose feces from China and carried *bla*_CTX-M-55_ gene [74], ST683 isolate was previously reported in human carbapenem-resistant *E. coli*, which was resistant to both cefotaxime and ceftazidime [75]. ST442 has been reported to be associated with Shiga toxin-producing *E. coli* [76], and ST2161 has been reported in fosfomycin-resistant *E. coli* from humans [77]. As far as we know, ST469, ST2854, and ST4040 have not been reported in ESBL-EC. Our results indicated that ESBL-EC isolates from captive primates exhibited diverse clonal lineages, which may be attributed to cross-infections among different animal species, as well as between humans and captive primates.’ (Tracked Version Line 378-387).

**Response to Reviewer 2 Comments**

**Response to Reviewer:**

Thank you for reading the manuscript carefully and providing many thoughtful comments, which will significantly improve the quality of the manuscript. We have carefully revised the manuscript in accordance with your comments (Tracked Version). Detailed responses are as follows:

**Reviewer #2:**

**Comment 1:** In the abstract you have to mention the studied samples, their number, their origins, region of the study. The aim not mentioned, the experiment were not mentioned. Perhaps you are limited by the number of words that must in the abstract but few data are needed to understand the work by learning the abstract.

**Response 1:** Thanks for your comments, we have improved the abstract section. We have added research objectives, sample information such as sources, quantities, and experimental methods to the abstract in our revised manuscript (The rapid spread of extended-spectrum β-lactamases (ESBLs)-producing *Escherichia coli* (ESBL-EC) around the world has become a significant challenge for humans and animals. In this study, we aimed to examine the characteristics and horizontal gene transfer (HGT) capacity of ESBL-EC derived from captive primates. We screened for ESBL-EC among a total of 444 multidrug-resistant (MDR) *E. coli* strains isolated from 13 zoos in China using double-disk test. ESBL genes, mobile genetic elements (MGEs), and virulence-associated genes (VAGs) in ESBL-EC were detected through polymerase chain reaction (PCR). Furthermore, conjugation experiments were conducted to examine the HGT capacity of ESBL-EC, and the population structure (phylogenetic groups and MLST) was determined.) (Tracked Version Line 6-12).

**Comment 2:** Abstract, line 19, write ‘...The population structure analysis showed that the phylogroup B1 and ST2161 were the most prevalent...’

**Response 2:** Thanks for your comment, we have corrected the sentence in the abstract section in our revised manuscript (Tracked Version Line 26-27).

**Comment 3:** Line 20 you wrote ‘ESBL-EC derived from captive primates pose a potential threat to animals, the environment, and humans through horizontal gene transfer.’ In reality the threat is by direct contact with colonized primates or by environmental pollution, so horizontal transfer is not a strict threat, perhaps genetic transfer is more implicated in the transfer between several genera colonizing primate. Take this in mind and try to modify this sentence in the abstract and this idea in the remaining part of your manuscript.

**Response 3:** Thank you for your comments. We have revised the manuscript to make the expression correct and scientific in our revised manuscript. Our revisions are as follows: ESBL-EC poses a potential threat to captive primates and may spread to other animals, humans, and the environment. It is imperative to implement measures to prevent the transmission of ESBL-EC among captive primates (Tracked Version Line28-30); ESBL-EC isolates may pose a serious threat not only to captive primates but also to other animals, humans, and the environment through cloning and direct contact (Tracked Version Line363-365).

**Comment 4:** In general we say ‘Mobile genetic element(s) (MGE)’ not ‘Mobile gene element (MGE)’ so modify this in the keywords and in the manuscript.

**Response 4:** Thanks for your comment, we have corrected the problem and made corrections to the errors elsewhere in our revised manuscript.

**Comment 5:** Write ‘Keywords: Captive primate; Escherichia coli; Extended‐spectrum β‐lactamase (ESBL), Virulence-associated gene (VAG); Mobile genetic elements (MGEs); Horizontal gene transfer (HGT).

**Response 5:** Thanks for your comments, we have adjusted the order of keywords in our revised manuscript (**Keywords:** Captive primate; *Escherichia coli***;** Extended‐spectrum β‐lactamase (ESBL); Virulence-associated gene (VAG); Mobile genetic element (MGE); Horizontal gene transfer (HGT)) (Tracked Version Line34-37).

**Comment 6:** Line 30, write ‘...Antimicrobial resistant (AMR) E. coli poses a threat to animal............ The production of extended-spectrum β-lactamases (ESBLs) by E. coli represents the primary mechanism responsible for the treatment failure by β-lactam antibiotics (5). The ESBL-producing E. coli (ESBL-EC) is one of the most important pathogens at the One...... CTX-Ms are the most predominant types of ESBLs which are encoded by several blaCTX-M type genes (7).’

**Response 6:** Thank you for your comments, we have checked and corrected the sentence in our revised manuscript (Antimicrobial resistant (AMR) *E. coli* poses a threat to animal and human health [3, 4]. The production of extended-spectrum β-lactamases (ESBLs) by *E. coli* represents the primary mechanism responsible for the treatment failure by β-lactam antibiotics [5]. The ESBL-producing *E. coli* (ESBL-EC) is one of the most important pathogens at the One Health interface [6]. Current ESBLs are mainly encoded by *bla*_CTX-M_, *bla*_TEM_*, bla*_SHV_ and *bla*_OXA_ genes. CTX-Ms are the most predominant types of ESBLs which are encoded by several *bla*_CTX-M_ type genes [7]) (Tracked Version Line41-48).

**Comment 7:** In all the manuscript including the abstract the name of bacteria must be in italic

**Response 7:** Thank you for your comments, we have carefully reviewed the manuscript and italicized the bacterial names.

**Comment 8:** Please verify the list of references, you need not to write words with capital letters, see the guideline of the journal and a recent article published in the journal. For example change ‘Negeri AA, Mamo H, Gurung JM, Firoj Mahmud AKM, Fällman M, Seyoum ET, et al. Antimicrobial Resistance Profiling and Molecular Epidemiological Analysis of Extended Spectrum β-Lactamases Produced by Extraintestinal Invasive Escherichia coli Isolates From Ethiopia: The Presence of International High-Risk Clones ST131 and ST410 Revealed. Front Microbiol. 2021;12:706846.’ BY ‘‘Negeri AA, Mamo H, Gurung JM, Firoj Mahmud AKM, Fällman M, Seyoum ET, et al. Antimicrobial resistance profiling and molecular epidemiological analysis of extended spectrum β-lactamases produced by extraintestinal invasive Escherichia coli isolates from Ethiopia: The presence of international high-risk clones ST131 and ST410 revealed. Front Microbiol. 2021;12:706846.’ VERIFY ALL REFERENCES.

**Response 8:** Thank you for your comments. We have carefully checked and revised our reference section, correcting some formatting errors. And we have revised the capitalization of the first letters in the reference section to ensure that the references comply with the journal's standards.

**Comment 9:** In this sentence ‘ESBL-EC has been frequently reported in captive and wild animals, such as giant pandas, ostriches 41 and white storks (12-15).’ YOU CAN ADD THIS REFERENCE AS REVIEW ARTICLE: Abbassi MS, Badi S, Lengliz S, Mansouri R, Salah H, Hynds P. Hiding in plain sight-wildlife as a neglected reservoir and pathway for the spread of antimicrobial resistance: a narrative review. FEMS Microbiol Ecol. 2022;98:fiac045.

**Response 9:** Thanks for your comments, we have added this reference in our revised manuscript, which improves the manuscript for the key information contained (Tracked Version Line55).

**Comment 10:** Line 44, write ‘In 2022, Zeng et al (18) isolated eight ESBL-EC strains from captive primates, all of which carried the blaCTX-M gene. In 2024, Bazalar-Gonzales et al (19) isolated seven ESBL-EC strains from captive and semi-captive primates, three blaCTM-X variants (blaCTX-M-15, blaCTX-M-55 and blaCTX-M-65) were identified.

**Response 10:** Thank you for your comments, we have corrected the sentence in our revised manuscript (In 2022, Zeng et al [19] isolated eight ESBL-EC strains from captive primates, all of which carried the *bla*_CTX-M_ gene. In 2024, Bazalar-Gonzales et al [20] isolated seven ESBL-EC strains from captive and semi-captive primates, three *bla*_CTM-X_ variants (*bla*_CTX-M-15_, *bla*_CTX-M-55_ and *bla*_CTX-M-65_) were identified) (Tracked Version Line59-63).

**Comment 11:** Line 57, write ‘Insertion sequences (ISs) have been frequently reported to be associated with the transfer of ESBLs genes, for example, ISEcp1 and IS26 have......... Because of this, blaCTX-M has rapidly become the most common ESBLs gene transferred by MGEs (26, 27)............. enhanced fitness of these emerging CTX-M-harboring strains (30, 31).’

**Response 11:** Thank you for your comments. We have corrected the problems in the sentence to ensure the correctness of the sentence in our revised manuscript (Insertion sequences (ISs) have been frequently reported to be associated with the transfer of ESBL genes, for example, *ISEcp1* and *IS26* have been widely reported to be associated with *bla*_CTX-M_ gene transmission. Because of this, *bla*_CTX-M_ has rapidly become the most common ESBL genes transferred by MGEs [27, 28]. Additionally, plasmids were also reported to carry various VAGs and to be associated with the transfer of VAGs, for example, IncF plasmid was the most reported virulence plasmid [29, 30]. Previous studies showed that ESBL-EC also harbored a range of VAGs, which may help to the enhanced fitness of these emerging CTX-M-harboring strains [31, 32].) (Tracked Version Line73-81).

**Comment 12:** Write ‘2.3 Screening of ESBL genes, VAGs and MGEs from ESBL-EC isolates’.

**Response 12:** Thank you for your comments. We have corrected the problem in our revised manuscript (2.3 Screening of ESBL genes, VAGs and MGEs from ESBL-EC isolates) (Tracked Version Line107-108).

**Comment 13:** The occurrence of the following mobile MGEs were investigated: ISEcp1, IS26, IS903, ISCR1, ISCR3/14, traA, trbC, traF, tnpU, tnpA/Tn21, tnsA, merA, tnp513, IS1133, ISpa7, ISaba1, ISkpn6, ISkpn7......... for the PCR primers were presented in Table S1.’

**Response 13:** Thank you for your comments, we have revised the sentence in our revised manuscript (The occurrence of the following MGEs were investigated: *ISEcp1, IS26, IS903, ISCR1, ISCR3/14, traA, trbC, traF, tnpU, tnpA/Tn21, tnsA, merA, tnp513, IS1133, ISpa7, ISaba1,* *ISkpn6* and *ISkpn7.* The sequences and conditions for the PCR primers were presented in S1 Table) (Tracked Version Line116-119).

**Comment 14:** PLEASE PROVIDE the reference of the conjugation experiment.

**Response 14:** Thanks for your comments, we have added reference for conjugation experiment in our revised manuscript (Transfer frequency was determined by calculating the ratio of the number of transconjugants to the number of recipient cells involved in the experiment [35]) (Tracked Version Line138).

Reference:

Zhang S, Guo X, Wang Y, Zhong Z, Wang M, Jia R, et al. Implications of different waterfowl farming on cephalosporin resistance: Investigating the role of *bla*(CTX-M-55). Poultry science. 2023;102(10):102929.

**Comment 15:** Write ‘’In this study, the sodium azide- resistant E. coli J53 was used...... Those capable of growing on Luria-Bertani (LB) agar supplemented with CTX (4 mg/mL) and sodium azide (150 mg/mL)..’

**Response 15:** Thank you for your comment, we have corrected the problem in our revised manuscript (In this study, the sodium azide-resistant *E. coli* J53 was used as the recipient strain to evaluate the conjugation and transfer ability of ESBL-EC. Those colonies capable of growing on Luria-Bertani (LB) agar supplemented with CTX (4 mg/mL) and sodium aside (100 mg/mL) were considered transconjugants) (Tracked Version Line131).

**Comment 16:** Please this sentence is not clear, improve it ‘The dilution factor (30 to 300 single colonies on solid medium) was determined experimentally.’

**Response 16:** Thank you for your comments. We have corrected and improved the sentence to make the sentence correct in our revised manuscript (Select the culture plates at appropriate dilution gradients for colony counting (where the number of colonies ranges from 30 to 300)) (Tracked Version Line131).

**Comment 17:** Write ‘.....sulfonamides (sul1, sul2, sul3), quinolones/fluoroquinolones (qnrS, oqxAB), chlormphenicol/phenicol (flor, cmlA, cat1) … We then examined whether the transconjugants carry these same genes. In addition, VAGs,..... recognize K, B/O, FrepB, FIB, W, I1, FIA, FIIA, N, T, HI2, X, Y, FIC, A/C, L/M, P and HI1. Primers and reaction conditions were listed in table S1.’

**Response 17:** Thank you for your comments. We have corrected the errors in drug names and other issues in our revised manuscript (sulfonamides *(sul1*, *sul2*, *sul3*), quinolones/fluoroquinolones (*qnrS*, *oqxAB*), chloramphenicol/phenicol (*flor*, *cmlA*, *cat1*), and aminoglycosides (*aacC2*, *aphA3*) [34]. We then examined whether the transconjugants carry these same genes. In addition, VAGs, and MGEs were detected in transconjugants. Plasmid incompatibility groups were detected in bacteria (donor) and their transconjugants as described previously [36]. In brief, eighteen pairs of primers were designed to recognize K, B/O, FrepB, FIB, W, I1, FIA, FIIA, N, T, HI2, X, Y, FIC, A/C, L/M, P and HI1…) (Tracked Version Line140-147).

**Comment 18:** Write ‘Briefly, seven housekeeping genes（purA, recA, gyrB, icd, mdh, adk and fumC） were investigated by PCR, the obtained DNA fragments were sequenced and uploaded to the website (PLEASE INDICATE THE NAME OF THE WEBSITE) to obtain the allele number, and the sequence type (ST)......... Primers and reaction conditions are shown in table S1’.

**Response 18:** Thank you for your comments. We have corrected the sentence and added the website name in our revised manuscript (Briefly, seven housekeeping genes (*purA*, *recA* *gyrB*, *icd*, *mdh*, *adk* and *fumC*) were investigated by PCR, the obtained DNA fragments were sequenced and uploaded to the website (https://pubmlst.org) to obtain the allele number, and the sequence type (ST) was obtained through the 7 allele numbers [38, 39]. Primers and reaction…) (Tracked Version Line153-158).

**Comment 19:** Write ‘..As shown in figure 1 and table S2, the detection........

**Response 19:** Thank you for your comments. We carefully reviewed the journal guidance([https://journals.plos.org/plosone/s/file?id=wjVg/PLOSOne_formatting_sample_main_body.pdf](https://track.editorialmanager.com/CL0/https:%2F%2Fjournals.plos.org%2Fplosone%2Fs%2Ffile%3Fid=wjVg%2FPLOSOne_formatting_sample_main_body.pdf/1/010f0194818870ce-75590e06-b46e-41dc-946c-cd81add20696-000000/XGd1OPkOrJEXeAdoDFXA-WkeJY5PH6MyIPApoJFNv74=194)) and use of Fig 1, S2 Table for the naming of figures and supporting tables was appropriate according to the journal guidance, and we also checked the figures and tables and supporting tables elsewhere to ensure that the naming was correct.

**Comment 20:** Please in line 143-144, indicate if these TEM variants are ESBLor penicillinase; if you prefer to say this in the discussion no problem, but you should say this.

**Response 20:** Thanks for your comments, we have noted the properties of the enzymes to which this gene belongs in the results section in our revised manuscript (Three variants of *bla*_TEM_ were identified, with *bla*_TEM-1_ (encoding penicillinase) being the most prevalent (28.99%, 20/69), followed by *bla*_TEM-135_ (encoding penicillinase) (10.14%, 7/69), and *bla*_TEM-198_ (encoding ESBL) (15.94%, 11/69)) (Tracked Version Line180-182).

**Comment 21:** ‘As shown in Figure 2 and table 1, the most prevalent VAGs was fimC.....’

**Response 21:** Thank you for your comments, We carefully reviewed the journal guidance([https://journals.plos.org/plosone/s/file?id=wjVg/PLOSOne_formatting_sample_main_body.pdf](https://track.editorialmanager.com/CL0/https:%2F%2Fjournals.plos.org%2Fplosone%2Fs%2Ffile%3Fid=wjVg%2FPLOSOne_formatting_sample_main_body.pdf/1/010f0194818870ce-75590e06-b46e-41dc-946c-cd81add20696-000000/XGd1OPkOrJEXeAdoDFXA-WkeJY5PH6MyIPApoJFNv74=194)) and use of Fig 1, S2 Table, Table 1 for the naming of figures and supporting tables and tabes was appropriate according to the journal guidance, and we also checked the figures and tables and supporting tables elsewhere to ensure that the naming was correct.

**Comment 22:** Write ‘ The proportion of strains carrying three VAGs was the most prevalent (20.29 %, 14/69), followed by strains carrying six (6/69; 18.84 % ) and five (5/69; 13.04%) VAGs.

**Response 22:** Thanks for your comment, we have corrected the sentence in our revised manuscript (The proportion of strains carrying three VAGs was the most prevalent (20.29%, 14/69), followed by strains carrying six (6/69 18.84%) and five (5/69 13.04%) VAGs) (Tracked Version Line192-196).

**Comment 23:** I changed this sentence ‘The strain carrying the greatest number of virulence genes was 10, accounting 158 for 4.35 % (3/69) of the total (Fig 2B).’ By ‘Interestingly, 3 strains (4.35%) harbored ten VRGs (Figure 2B).’ THE SENTENCE WAS NOT CLEAR, PLEASE VERIFY AND MAKE CORRECTION.

**Response 23:** Thank you for pointing out the problem, we have rewritten the sentence in accordance with your comment in our revised manuscript, so that the sentence is more concise and easier to understand (Tracked Version Line196-197).

**Comment 24:** In material and method you mentioned that you searched 18 MGEs including ISEcp1, IS26, IS903, ISCR1, ISCR3/14, traA, trbC, traF, tnpU, tnpA/Tn21, tnsA, merA, tnp513, IS1133, ISpa7, ISaba1, ISkpn6, ISkpn7. But in the list of primers there are several primers missed for example those of ISkpn6, ISkpn7, traA, .... PLEASE VERIFY and add them in the table of primers. PLEASE VERIFY ALSO THIS IN THE SUPPLEMENTARY file, TABLE S1.

**Response 24:** Thank you for pointing out the problem. We have checked table S1 and added the missing primers (*ISaba1*, *ISkpn6*, *ISkpn7*) to S1 Table.

**Comment 25:** Write ‘...with IS26+ISEcp1 being the most prevalent (13/69; 18.84 %). ......... MGE, except for ESBLs-E.coli 55 and ESBLs-E.c56.’

**Response 25:** Thanks for your comment, we have corrected the problem in our revised manuscript (…with *IS26*+*ISEcp1* being the most prevalent (13/69, 18.84%). A total of 97.10% (67/69) ESBL-EC isolates exhibited the presence of at least one MGE, except for ESBLs-*E. coli* 55 and ESBLs-*E. coli* 56.) (Tracked Version Line20).

**Comment 26:** Line 174, please verify, there some combinations are repeated twice, for example blaCTX-M-65/tnpA/Tn21, blaCTX-M-65/IS1133. I modifiy as follows: ‘(blaCTX-M-15/ISEcp1, blaCTX-M-15/tnsA, blaCTX-M-55/trbC, blaCTX-M-55/merA, blaCTX-M-55/IS26, blaCTX-M-55/ISCR3/14, blaCTX-M-65/tnpA/Tn21, blaCTX-M-65/IS1133, blaCTX-M-55/trbC, , blaCTX-M-65/tnpA/Tnp21, blaCTX-M-65/IS1133, blaTEM-1/trbC, blaTEM-1/ISEcp1)’. ALSO VERIFY THESE COMBINATION: ‘Seven 175 pairs of negative correlations were detected (blaCTX-M-15/trbC, blaCTX-M-15/merA, blaCTX-M176 15/tnpA/Tn21, blaCTX-M-15/IS26, blaTEM-135/ISEcp1, blaCTX-M-15/trbC, blaCTX-M-15/merA, blaTEM-M177 15/tnpA/Tn21, blaCTX-M-15/IS26, blaTEM-135/ISEcp1, blaCTX-M-55/ISEcp1, blaCTX-M-55/tnsA).’ Verify and correct.

**Response 26:** Thank you for pointing out the problem carefully, we have checked and revised the manuscript to ensure there are no errors ((*bla*_CTX-M-15_/*ISEcp1*, *bla*_CTX-M-15_/*tnsA*, *bla*_CTX-M-65_/*tnpA/Tn21*, *bla*_CTX-M-65_/*IS1133*, *bla*_CTX-M-55_/*trbC*, *bla*_CTX-M-55_/*merA*, *bla*_CTX-M-55_/*IS26*, *bla*_CTX-M-55_/*ISCR3/14*, *bla*_TEM-1_/*trbC*, *bla*_TEM-1_/*IS26*, *bla*_TEM-135_/*ISCR3/4*). Seven pairs of negative correlations were detected (*bla*_CTX-M-15_/*trbC*, *bla*_CTX-M-15_/*merA*, *bla*_CTX-M-15_/*tnpA/Tn21*, *bla*_CTX-M-15_/*IS26*, *bla*_TEM-135_/*ISEcp1*, *bla*_CTX-M-55_/*ISEcp1*, *bla*_CTX-M-55_/*tnsA*)) (Tracked Version Line214-220).

**Comment 27:** What mean ‘negative correlations’ please explain it by single sentence in this sentence:’.. Seven pairs of negative correlations were detected (blaCTX-M-15/trbC..’

**Response 27:** Thank you for your comment, we have revised the manuscript to add possible reasons in the discussion section as follows: “Negative correlations between ESBL genes and MGEs were also detected. These correlations may be attributed to the presence of specific combinations of ESBL genes with MGEs, but the specific reasons for these correlations need to be further studied [58, 59]” (Tracked Version Line326-329).

**Comment 28:** Line 182, you wrote ‘Our result showed that 28 of 69 ESBL-EC isolates were successfully transferred to the recipient strain J53’ PLEASE what was transferred? Cefotaxime resistance? ESBL phenotype?. Improve the sentence.

**Response 28:** Thank you for pointing out the problem. Both ESBL phenotype and ESBL gene (*bla*_CTX-M_) were detected in transconjugants. We have modified this place to make the expression correct and scientific in our revised manuscript (Our results showed that 28 of 69 ESBL-EC isolates were successfully transferred to the recipient strain J53, and all transconjugants exhibited the ESBL phenotype and were positive for ESBL genes (*bla*_CTX-M_)) (Tracked Version Line226-227).

**Comment 29:** Write ‘The conjugational transfer frequency of the 28 ESBL-EC strains ranging from 1.18×10- 8 to 2.14×10-4 tranconjugants/recipient strain.’

**Response 29:** Thank you for pointing out the problem, and we have corrected it in our revised manuscript (The conjugational transfer frequency of the 28 ESBL-EC strains ranging from 1.18×10^-8^ to 2.14×10^-4^ transconjugants/recipient strain) (Tracked Version Line228).

**Comment 30:** Line 197, write ‘..MLST analysis showed a high diversity of STs (sequence type), and 11 different STs were observed: ST2161 (10/28; 35.71 %), followed by ST442 (7/28; 25.00 %), ST2854 200 (2/28; 7.14 %) and ST683 (2/28; 7.14 %).’

**Response 30:** Thanks for your comments, we have revised the sentence t in our revised manuscript (MLST analysis showed a high diversity of STs (sequence type), and 11 different STs were observed: ST2161 (10/28, 35.71%), followed by ST442 (7/28, 25.00%), ST2854 (2/28, 7.14%) and ST683 (2/28, 7.14%)) (Tracked Version Line243-246).

**Comment 31:** Please in table S1, change Amide alcohols by ‘chlormphenicol/phenicol’

**Response 31:** Thank you for pointing out the problem. We have modified S1 table.

**Comment 32:** In discussion line 205, the following sentence is not correct, you mean thing but you write other thing ‘However, ESBLs produced by Enterobacteriaceae are ineffective against β-lactam drugs (39).’ I propose :’ β-lactams are the most prescribed antibiotics worldwide. However, β-lactam antibiotics are ineffective against ESBLs-producing Enterobacteriaceae (39).’

**Response 32:** Thanks for your kind advice, we did misrepresent what we wanted to say, we have revised the sentence to make it correct in our revised manuscript (β-lactams are the most prescribed antibiotics worldwide. However, β-lactams antibiotics are ineffective against ESBLs-producing *Enterobacteriaceae*) (Tracked Version Line252-253).

**Comment 33:** Line 216, improve this sentence, it is not clear ‘The prevalence of the blaCTX-M-55 in our present study is 214 higher than that of another variant of blaCTX-M’.

**Response 33:** Thank you for your comments, we have revised the sentence to make it clear in our revised manuscript (In our study, the prevalence of *bla*_CTX-M-55_ was also higher than that of other detected *bla*_CTX-M_ variants, including *bla*_CTX-M-65_, *bla*_CTX-M-14_, and *bla*_CTX-M-3_) (Tracked Version Line261-263).

**Comment 34:** Line 219, please highlight that the CTX-M-55 is the most relevant CTX-M enzyme in China (please verify if it is relevant from animal and human isolates), I believe also in other Asian countries, in other European countries and Africa CTX-M-1 is relevant in animal but CTX-M-15 IN HUMAN ISOLATES. This is very important to say that the scenario in animal mirror the scenario (the epidemiology of ESBL enzymes) in human isolates. A large view of the epidemiology of ESBL enzymes is requested to understand the ways of ESBL transmission in Humn/Animal/environmental interface under the One Health approach.

**Response 34:** Thank you for your comments. Currently, *E. coli* carrying *bla*_CTX-M - 55_ has been widely reported in clinical patients and farm animals in China and is showing a rapidly increasing trend. Nowadays, CTX-M-55 is the most common CTX-M enzyme in animal-derived *E. coli* in China. Although CTX-M-14 is currently the most common in human in China, CTX-M-55 has surpassed CTX-M-15 to become the second-most common CTX-M enzyme. According to previous reports, ESBL-EC tends to spread from animals to humans through the food and companion animals. Moreover, there are also reports of transmission to humans via wild birds. Therefore, the reports of CTX-M-55 in human clinical cases are likely to continue to increase, and CTX-M-55 may become the most common CTX-M enzyme in human in the future. Thank you for your valuable suggestions. We have already highlight in the discussion that CTX - M - 55 is currently the most relevant enzyme in China (Currently, CTX-M-55 is the most relevant CTX-M enzyme in China. It is the most frequently reported enzyme in animals, and is reported to have an increasing trend in human clinical isolates [44].) (Tracked Version Line266-268).

Reference:

Yang, J. T., Zhang, L. J., Lu, Y., Zhang, R. M., & Jiang, H. X. (2023). Genomic Insights into Global *bla*_CTX-M-55_-Positive Escherichia coli Epidemiology and Transmission Characteristics. *Microbiology spectrum*,*11*(4), e0108923.

**Comment 35:** Write ‘MGEs play an important role in the transmission of ESBL-producing capability, which is mediate by the ESBL genes (21, 22)’

**Response 35:** Thanks for your comment, we have corrected the issue in our revised manuscript (MGEs play an important role in the transmission of ESBL-producing capability, which is mediate by the ESBL genes) (Tracked Version Line274).

**Comment 36:** In all your manuscript you have to write ‘ESBL genes’ not ‘ESBLs genes’, also not ‘ESBL genes’

**Response 36:** Thanks for your comments, we have carefully examined all parts of the manuscript (including Figure legends, Supplementary table) and corrected the incorrect writing.

**Comment 37:** Xorrect this sentence by ‘Our present study showed that qnrS (quinolone/fluoroquinolone resistance gene), tetA (tetracycline resistance gene), sul2 (sulfonamides resistance gene), cmlA and floR (chlormphenicol/phenicol resistance genes)’

**Response 37:** Thank you for pointing out the error, we have corrected it in our revised manuscript (Our present study showed that *qnrS* (quinolone/fluoroquinolone resistance gene), *tetA* (tetracycline resistance gene), *sul2* (sulfonamides resistance gene), *cmlA* and *floR* (chloramphenicol/phenicol resistance genes)) (Tracked Version Line351-354).

**Comment 38:** Line 285, you performed PCR of virulence gene in the recipient strain?, I believe that this strain harbor papA and fimC; if you did not so you cannot say ‘Notably, all papA and fimC were detected in the corresponding transconjugants.’ Since that two gene can already exist in the recipient strain especially when taken in mind that these genes encode simple fimbrae, very common in E. coli isolates. Verify this point.

**Response 38:** Thank you for your comments. We did not perform PCR on J53 for *papA* and *fimC*, we have removed this sentence to make our descriptions accurate and scientific in our revised manuscript (Tracked Version Line359).
